# Adherence and Wearing Time of Prescribed Footwear among People at Risk of Diabetes-Related Foot Ulcers: Which Measure to Use?

**DOI:** 10.3390/s23031648

**Published:** 2023-02-02

**Authors:** Gustav Jarl, Chantal M. Hulshof, Tessa E. Busch-Westbroek, Sicco A. Bus, Jaap J. van Netten

**Affiliations:** 1 Department of Prosthetics and Orthotics, Faculty of Medicine and Health, Örebro University, Örebro, Sweden; 2 University Health Care Research Center, Faculty of Medicine and Health, Örebro University, Örebro, Sweden; 3 Department of Rehabilitation Medicine, Amsterdam UMC, University of Amsterdam, Meibergdreef 9, 1105 AZ Amsterdam, The Netherlands; 4 Amsterdam Movement Sciences, Ageing & Vitality and Rehabilitation & Development, Amsterdam, The Netherlands

**Keywords:** diabetic foot, foot ulcer, treatment adherence and compliance, patient compliance, footwear, shoes, validation study

## Abstract

Adherence to prescribed footwear is essential to prevent diabetes-related foot ulcers. The aim was to compare different measures of adherence and wearing time of prescribed footwear with a reference adherence measure, among people with diabetes at high risk of foot ulceration. We followed 53 participants for 7 consecutive days. A temperature sensor measured wearing time of prescribed footwear and a triaxial accelerometer assessed weight-bearing activities. Subjective wearing time was self-reported. Reference adherence measure was proportion of weight-bearing time prescribed footwear was worn. We calculated Spearman’s correlation coefficients, kappa coefficients, and areas under the curve (AUC) for the association between the reference measure and other measures of adherence and wearing time. Proportion of daily steps with prescribed footwear worn had a very strong association (r = 0.96, Κ = 0.93; AUC: 0.96–1.00), objective wearing time had a strong association (r = 0.91, Κ = 0.85, AUC: 0.89–0.99), and subjective wearing time had a weak association (r = 0.42, Κ = 0.38, AUC: 0.67–0.81) with the reference measure. Objectively measured proportion of daily steps with prescribed footwear is a valid measure of footwear adherence. Objective wearing time is reasonably valid, and may be used in clinical practice and for long-term measurements. Subjective wearing time is not recommended to be used.

## 1. Introduction

Adherence to wearing prescribed footwear by people with diabetes mellitus at risk of foot ulceration is important in the prevention of foot ulcers. Of people with diabetes, 19–34% develop a diabetes-related foot ulcer during their life time [1]. International [2] and national evidence-based guidelines [3,4,5] recommend the use of therapeutic footwear to reduce this risk. However, several studies have reported that patients’ adherence to wearing therapeutic footwear is often low, with patients wearing the footwear for approximately 50% of waking day time [6,7] or 70% of daily number of steps [8], which may contribute to the high recurrence rate of foot ulcers [1,9,10]. Researchers have tried to address this by investigating predictors of adherence [6,11,12] and evaluating interventions to improve adherence [13]. However, studies on different aspects of adherence are difficult to compare and synthesize as the studies have used different methods, both objective and subjective, to assess adherence and wearing time of prescribed footwear [14,15].

The preferred definition of adherence is wearing time of prescribed footwear during and as percentage of weight-bearing activities, as weight-bearing activities typically expose the feet to risk of developing foot ulcers [14,16]. This definition of adherence implies that the reference standard for adherence measurement should include simultaneous and objective measurement of wearing time of prescribed footwear and weight-bearing activities. This can be achieved by securing a sensor in the footwear and an activity monitor on the body, which measures type and duration of weight-bearing activities such as walking and standing [8,10,13,17,18,19,20,21,22,23]. Some studies have used pedometers that record number of steps; however, this measure does not record other weight-bearing activities, such as standing duration, which also puts stress on the feet [24]. Other studies have only measured wearing time of the footwear, without assessing weight-bearing activities of the person; in these studies it is unknown how much the footwear was worn during weight-bearing activities that exposed the feet to risk of foot ulcers [25,26]. In addition to these objective methods, a number of studies have used subjective methods, such as structured interviews and questionnaires, to estimate wearing time [14]. Typically, patients answer a multiple-choice question regarding the daily number of hours (or the proportion of daytime) they use their prescribed footwear. In some studies, this self-reported hours of daily use is weighted by the self-reported number of days the footwear is worn each week [7,14,27,28]. However, each study usually uses only one method to measure adherence or wearing time, which is why we cannot know if the different measures are comparable. We are aware of only one study comparing different measures, reporting a strong correlation (r = 0.87) between objectively measured wearing time and objectively measured proportion of steps the prescribed footwear was worn [8]. No study has investigated the validity of different measures of adherence and wearing time in comparison with the proportion of weight-bearing activity time that prescribed footwear is worn, the reference standard for adherence measurement. Thus, the aim of the study was to compare different objective and subjective measures of adherence and wearing time of prescribed footwear to the reference adherence measure, among people at risk of diabetes-related foot ulcers.

## 2. Materials and Methods

### 2.1. Participants

Participants were recruited at the two locations of Amsterdam UMC and at podiatry practice Voeten op Texel, in the Netherlands, as part of the study DIALOAD (https://www.trialregister.nl/trial/8839, accessed on 15 December 2022). The DIALOAD is a prospective observational cohort study in which people at high risk of diabetic foot ulceration are followed for 12 months with the aim to unravel biomechanical and behavioral mechanisms of foot ulceration. During August 2020–May 2022, high-risk people visiting the outpatient clinic consultation hours were consecutively screened for eligibility to participate in the study. One hundred three potential participants were informed about the study and asked for interest to participate. Sixty-three people provided written informed consent to participate in the study, whereof three were excluded due to not meeting the criteria to be included, giving sixty people participating in the data collection. Inclusion criteria were age ≥ 18 years; diabetes mellitus type 1 or 2; recent history of a diabetes-related foot ulcer (<1 year); or forefoot/midfoot barefoot peak plantar pressure > 600 kPa, being ambulatory and loss of protective sensation (inability to feel a 10 g monofilament and tuning fork following criteria of the IWGDF guidelines [29]). Exclusion criteria were diabetes-related foot ulcer; open amputation site; active Charcot neuro-osteo arthropathy; or use of walking aid for full support and severe peripheral artery disease (WIfI grade 3 [30]).

### 2.2. Procedures and Data Collection

At study baseline, the participants underwent a physical examination and the participant with semi- or fully custom-made footwear answered the Monitor Orthopaedic Shoes questionnaire [28]. Weight-bearing activities were measured with a triaxial accelerometer for seven consecutive days after the baseline visit (DynaPort MoveMonitor, McRoberts, The Hague, The Netherlands) [31]. The accelerometer had to be worn in the middle of their back, at level L5, and could only be removed during water activities. It has a 100 Hz sampling frequency, ±6 g range, and 12-bit resolution. The accelerometer had to be worn ≥ 75% of 24 h [32], or ≥12 h if not worn at night [33]. To assess wearing time of prescribed footwear, a temperature sensor (Orthotimer, Rollerwerk, Balingen, Germany), validated in a previous study [26], was secured in the medial arch support of the prescribed footwear’s insole (and in a maximum of four pairs) of each participant [25]. One sensor was used per pair of footwear. The sensor was placed in the most appropriate shoe based on foot health, that is, presence of deformities, amputations, pre-signs of ulceration, and/or previous ulcer location. The sensor measured and stored time-stamped temperatures every 15 min. Wearing time was assessed for the same seven days during which the accelerometer was worn. At least four valid days of both activity and temperature data were required for the participant to be included in the analysis [32].

### 2.3. Measures of Adherence

Objectively measured proportion of weight-bearing time the prescribed footwear was worn was the reference standard, that is, the measure to which the other measures of adherence and wearing time were compared. Two objective and two subjective measures were compared to this reference standard. The reference standard and both objective measures were based on the data from the accelerometer and temperature sensor. The raw data from the accelerometer were categorized using the validated algorithms of the manufacturer into periods of walking, standing, shuffling, stair walking, lying, sitting, cycling, and nonwearing [34,35]. We defined walking, standing, shuffling, and stair walking as weight-bearing activities. The raw data from the temperature sensor were used to determine when the footwear was worn and not worn, using the adapted validated Groningen algorithm [25]. The first objective measure (“Proportion of steps”) was defined as the proportion of steps that the prescribed footwear was worn. The second objective measure (“Objective wearing time”) was defined as the average daily time that the prescribed footwear was worn. All objective adherence measures were obtained by using custom-written scripts in Matlab (R2021b, The MathWorks, Inc., Natick, MA, USA) and averaged over all valid days.

The two subjective measures were based on two questionnaire items asking for the number of hours (h) each day and number of days each week the prescribed footwear was worn [28]. Rating scales were >12, 8–12, 4–8, 1–4, and <1 h/day, and 6–7, 4–5, 2–3, 1, and 0 days/week, respectively. The first subjective measure (“Subjective wearing time”) consisted of the participant’s answer regarding the number of h/day the prescribed footwear was worn. The second subjective adherence measure (“Weighted subjective wearing time”) consisted of the median self-reported h/day multiplied with the median self-reported days/week divided by 7 (days) [7]. For example, if a participant answered “>12 h/day” and “6–7 days/week”, the average wearing time would be 14 × 6.5/7 = 13 h/day (assuming 16 h/day out of bed, “>12 h/day” was given the median value of 14).

### 2.4. Statistical Analysis

Data distributions were first tested for normality using the Shapiro–Wilk test. As neither the reference measure (*p* = 0.001) nor any of the other four measures of adherence and wearing time (*p*-values < 0.001–0.019) were normally distributed, Spearman’s correlation coefficient was calculated for all correlations. First, we calculated the correlation between the reference adherence measure and each of the other measures. We also calculated the correlation between the remaining pairs of measures. Correlation coefficients between 0.00–0.09 were considered negligible, 0.10–0.39 weak, 0.40–0.69 moderate, 0.70–0.89 strong, and 0.90–1.00 were considered very strong [36]. We then calculated the kappa coefficient (Κ) with quadratic weights between the reference measure and the other measures of adherence and wearing time. In this analysis, the reference measure and proportion of daily steps footwear was worn were categorized into 0–20%, >20–40%, >40–60%, >60–80%, and >80–100% adherence and proportion of steps footwear was worn, respectively. Objective wearing time was categorized according to the increments used as the rating scale for subjective wearing time, i.e., >12, 8–12, 4–8, 1–4, and <1 h/day. Kappa values in the range 0–0.20 were considered to reflect no agreement, 0.21–0.39 minimal agreement, 0.40–0.59 weak agreement, 0.60–0.79 moderate agreement, 0.80–0.90 strong agreement, and >0.90 almost perfect agreement [37]. Finally, we calculated the area under the curve (AUC) in the receiver operating characteristic (ROC) curve, and sensitivity, specificity, positive predictive value, and negative predictive value for each measure. In these analyses, we dichotomized participants into highly and lowly adherent according to the reference measure, using 60%, 70%, 80%, and 90% as cut-offs. An AUC of 1.0 indicates that the measure perfectly classifies participants as highly or lowly adherent. An AUC range 0.7–0.8 is considered acceptable, 0.8–0.9 is considered excellent, and >0.9 is considered outstanding [38]; *p*-values < 0.05 and 95% confidence intervals not overlapping zero were considered to indicate statistical significance in all tests. We used the Vasserstat website (http://vassarstats.net/, accessed on 15 December 2022) to calculate the kappa coefficient and IBM SPSS Statistics for Windows, version 26.0 (Armonk, NY, USA: IBM Corp.) for all other analyses.

## 3. Results

Seven of the sixty participants were excluded after data collection due to missing temperature sensor data for one or more sensors (*n* = 3), less than four valid days of activity (*n* = 3) and no activity data due to lost accelerometer (*n* = 1). The 53 participants who were included in the analyses consisted of 43 men and 10 women with a mean age of 65.3 years; 81.1% of the participants had diabetes type 2, all but 2 participants had a history of foot ulcers and the average body mass index was close to 30. More characteristics of the participants can be found in Table 1.

**Figure 1:**
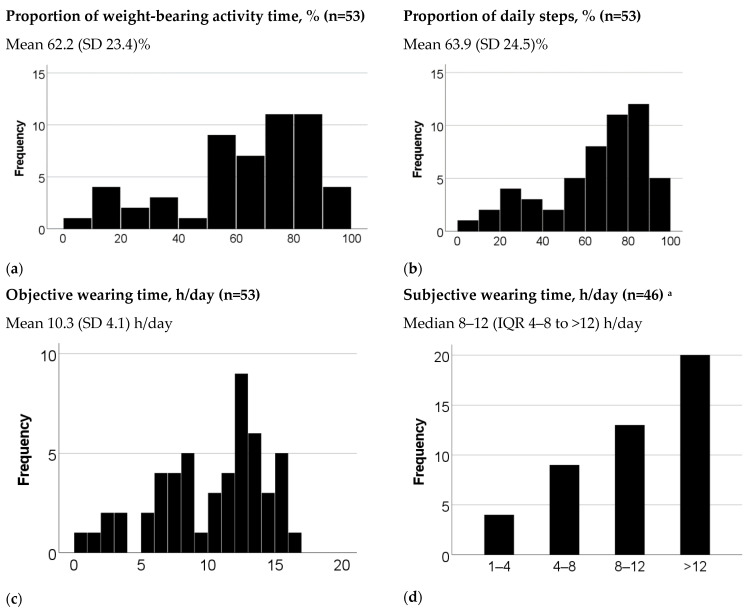

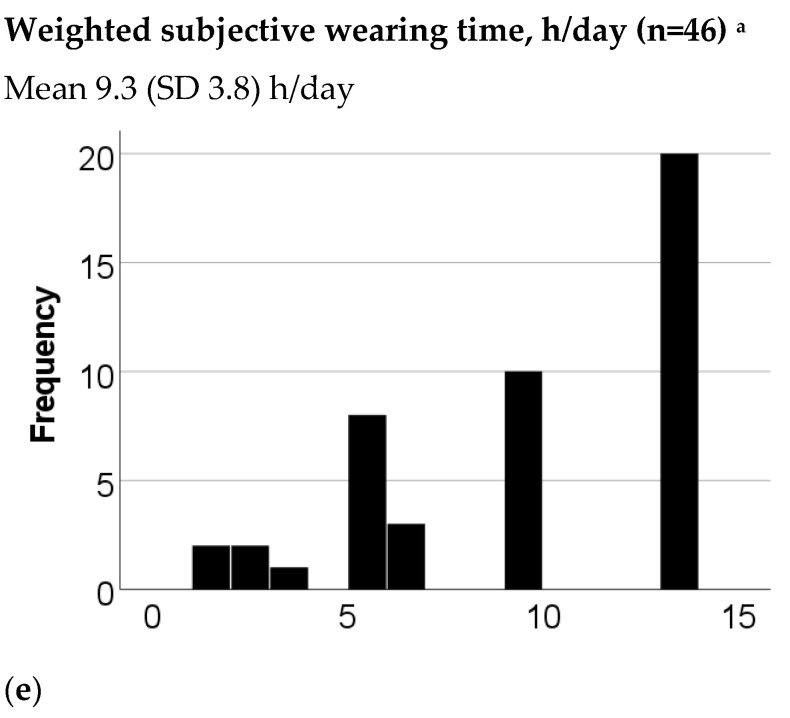
Summary of results on measures of adherence and wearing time. SD, standard deviation; IQR, interquartile range: ^a^ The six participants with prefabricated footwear did not answer the questions on subjective wearing time and one participant with fully custom-made footwear had missing answers.

Participants spent on average 3.5 h/day in weight-bearing activities (Table 1), and wore their prescribed footwear on average 62.2% of the weight-bearing activity time. Participants took on average 5835 daily steps and wore their prescribed footwear for 63.9% of these steps. Objective wearing time of prescribed footwear was on average 10.3 h/day. Median subjective wearing time was 8–12 h/day and average weighted subjective wearing time was 9.3 h/day (Figure 1).

**Figure 2:**
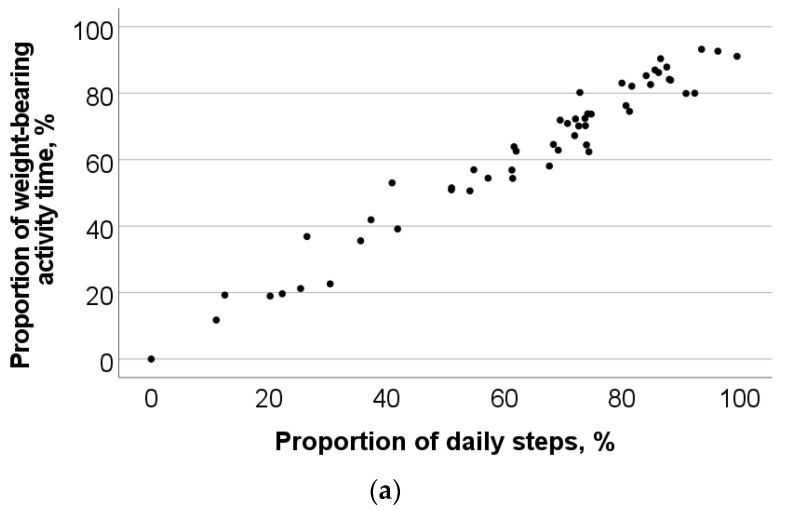

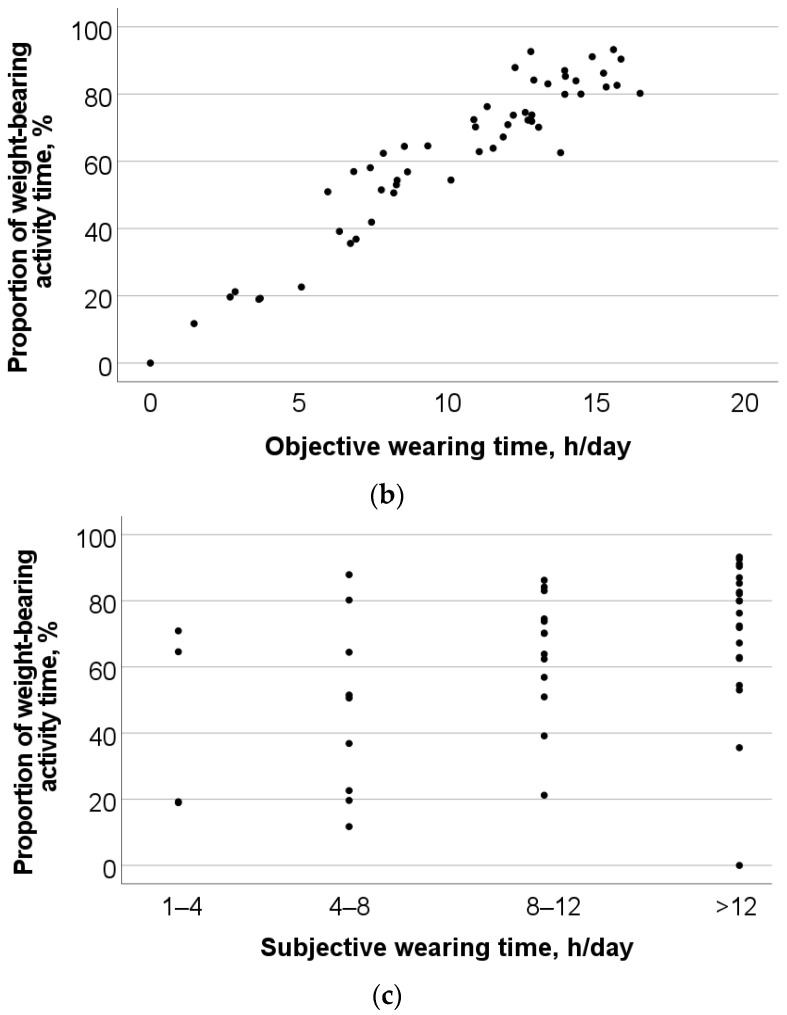
Scatter plots and Spearman’s correlation coefficients (r) for the associations with the reference measure: (**a**) r = 0.96 (*p* < 0.001), (**b**) r = 0.91 (*p* < 0.001), (**c**) r = 0.42 (*p* = 0.004).

The two subjective measures of wearing time had a very strong correlation, r = 0.99 (*p* < 0.001). Therefore, we decided to only include the (unweighted) subjective wearing time in the further analyses. Proportion of steps that the prescribed footwear was worn correlated very strongly (r = 0.96, *p* < 0.001) and objective wearing time correlated very strongly (r = 0.91, *p* < 0.001) with the reference measure (Figure 2). Subjective wearing time had a moderate correlation with the reference measure (r = 0.42, *p* = 0.004). Proportion of daily steps footwear was worn correlated strongly with objective wearing time (r = 0.87, *p* < 0.001) and moderately with subjective wearing time (r = 0.43, *p* = 0.003). Objective wearing time of footwear correlated moderately with subjective wearing time (r = 0.46, *p* = 0.001).

Proportion of daily steps prescribed footwear was worn had almost perfect agreement (Κ = 0.93, 95% CI not possible to calculate), objective wearing time had strong agreement (Κ = 0.85, 95% CI not possible to calculate), and subjective wearing time had minimal agreement (Κ = 0.38, 95% CI: 0.15–0.61) with the reference adherence measure. The AUC was outstanding for proportion of steps (0.96–1.00), ranged from excellent to outstanding for objective wearing time (0.89–0.99) and ranged from not acceptable to excellent for subjective wearing time (0.67–0.81), for the different cut-offs used to classify participants as highly or lowly adherent (Table 2).

Sensitivity was 93–100% for proportion of steps, 91–100% for objective wearing time, and 64–100% for subjective wearing time (Table 3). Specificity was 89–95% for proportion of steps, 69–100% for objective wearing time, and 43–66% for subjective wearing time. The positive predictive value was 44–97% for proportion of steps, 21–100% for objective wearing time, and 20–76% for subjective wearing time. The negative predictive value was 97–100% for proportion of steps, 87–100% for objective wearing time, and 62–100% for subjective wearing time.

For all cut-offs used to classify participants as highly and lowly adherent, the sensitivity and specificity values were equal or higher for proportion of steps than for the corresponding values for subjective wearing time (Table 3). In most cases, the sensitivity and specificity values for objective wearing time fell between the corresponding values for proportion of steps and subjective wearing time. For example, using ≥70% of weight-bearing activity time prescribed footwear is worn as cut-off for high adherence, wearing prescribed footwear for ≥69.4% of daily steps had a sensitivity of 100% and a specificity of 89%, an objective wearing time of ≥10.5 h/day had a sensitivity of 100% and a specificity of 85%, and a subjective wearing time of ≥8–12 h/day had a sensitivity of 87% and a specificity of 43%.

## 4. Discussion

This is the first study that focused on the comparison of different methods to measure adherence and wearing time of prescribed footwear among people at high risk of developing diabetes-related foot ulcers. As adherence to wearing prescribed footwear is essential to reduce the risk of foot ulcers [10], it is important to assess adherence in a valid way. We found that the reference measure of adherence, proportion of weight-bearing activity time that the prescribed footwear was worn, was very strongly associated with proportion of daily steps footwear was worn, strongly associated with objective wearing time, but only weakly associated with subjective wearing time. We used different cut-offs to dichotomize the participants into those with high and low adherence, as different cut-offs have been suggested in the literature [15]. However, the main results were not dependent on the particular cut-off chosen: proportion of daily steps was the most valid measure in all comparisons and subjective wearing time was the least valid measure.

These findings suggest that proportion of steps that prescribed footwear is worn is a valid estimate of proportion of weight-bearing activity time that the footwear is worn, in both research and clinical contexts. This implies that findings of studies using these two measures as outcomes are comparable, and that simple and less expensive activity monitors, e.g., validated pedometers, can be used to determine adherence [35]. The strong correlation of objective wearing time with the reference measure implies that wearing time may be valid to estimate proportion of weight-bearing activity time that the footwear is worn. Measuring wearing time only requires a sensor in the footwear and is therefore less burdensome to patients and less expensive than the other objective measures that require an additional activity monitor to be worn on the body [14]. In addition, wearing time sensors typically can record and store data for longer times than accelerometers, enabling long-term measurements. However, although objective wearing time may be useful to measure in clinical practice, the association with the reference measure of adherence may not be strong enough in all research contexts. Therefore, as the outcome measure, research studies on footwear adherence should preferably use proportion of steps or proportion of weight-bearing activity time prescribed footwear is worn.

The two subjective measures of wearing time of prescribed footwear correlated very strongly with each other. Therefore, we chose to only include one of them in further analyses, where we found that the correlations with the reference measure and the other two objective measures were weak. This suggests that these two subjective measures of wearing time are not valid to be used. This is an important finding as similar subjective measures of wearing time have frequently been used in research studies [11,14] and are often used in clinical practice. Because subjective methods are easy to use in any setting, further development and testing of other subjective measures than the ones tested in this study may provide a more valid subjective alternative. Potential subjective measures could include footwear wearing diaries or more elaborate questionnaires to estimate wearing of prescribed footwear from a number of different questions.

There is no gold standard measure of adherence [39]. The adherence measure used as reference in this study is based on the assumption that adherence is defined in terms of using prescribed footwear during all weight-bearing activities. The purpose of using prescribed footwear in people with diabetes is to protect the feet against all acute and chronic trauma that could trigger the development of a foot ulcer in the presence of predisposing risk factors, such as, loss of protective sensation, foot deformities, and peripheral artery disease [40]. Under the assumption that ulcer-inducing trauma can only occur during weight-bearing activities, such as standing and walking, it is reasonable to define adherence as the proportion of weight-bearing activity time prescribed footwear is used, and use the reference measure of this study as the method in which to compare other measures. However, for some patients, ulcer-inducing trauma may present outside weight-bearing activities. For example, for a patient sitting in a wheel-chair all day, adherence could be defined as the proportion of overall out-of-bed time the prescribed footwear is worn and, thus, objective wearing time is a more appropriate adherence measure to be used as reference. This has implications for the definition of adherence to prescribed footwear. The International Working Group on the Diabetic Foot defines adherence to offloading intervention as “the extent to which a person’s behavior corresponds with agreed recommendations for treatment from a health care provider, expressed as quantitatively as possible; usually defined as the proportion of time using the prescribed offloading intervention of the total time in which the intervention is prescribed to be used (e.g., % of the total weight bearing time that the patient was wearing the prescribed offloading device)” [41]. In the context of adherence to prescribed footwear to prevent diabetes-related foot ulcers, this definition may need to reflect all situations that include risk of ulcer-inducing trauma.

Strengths of the study were that different measures of footwear adherence and wearing time were compared in the same people, and validated algorithms were used to classify activities and determine when footwear was worn. Furthermore, we measured wearing time with temperature sensors in up to four pairs of footwear per participants. Although six participants had more than four pairs of prescribed footwear, we believe these are too few to have had any substantial impact on the results. A limitation of the study was the missing data on the subjective wearing time of prescribed footwear, which resulted in wide confidence intervals on the estimations of the AUC for subjective wearing time.

## 5. Conclusions

Objectively measured proportion of daily steps prescribed footwear is worn is a valid measure of footwear adherence. Objectively measured wearing time is reasonably valid, and may be used in clinical practice and for long-term measurements. The two subjective measures of wearing time are not recommended to be used.

## Figures and Tables

**Table 1:** Characteristics of study participants and daily activities with and without prescribed footwear.

Participants’ Characteristics			Mean (SD) or n (%)
Sex, men/women			43 (81.1)/10 (18.9)
Age, years			65.3 (9.4)
BMI			29.6 (5.6)
Diabetes type, type 1/2			10 (18.9)/43 (81.1)
Diabetes duration, years			18.9 (12.0)
HbA1c (n = 6 missing)	NGSP, %		7.7 (3.8)
	IFCC, mmol/mol		60.6 (17.9)
Foot deformities a	Absent		0
	Mild		2 (3.8)
	Moderate		45 (84.9)
	Severe		6 (11.3)
History of foot ulcer			51 (96.2)
Amputations b	No		33 (62.3)
	Smaller toes		8 (15.1)
	Hallux or more proximal partial foot		10 (18.9)
	Through or above ankle		2 (3.8)
Type of prescribed footwear	Prefabricated		6 (11.3)
	Semi-custom-made		14 (26.4)
	Fully custom-made		33 (62.3)
**Steps and weight-bearing activities with and without prescribed footwear**			
	With prescribed footwear	Total, with and without prescribed footwear	Proportion with prescribed footwear, %
Number of daily steps, mean (SD)	3678 (2784)	5835 (3731)	63.9 (24.5)
Number of hours of daily weight-bearing activity time, mean (SD)	2.2 (1.3)	3.5 (1.6)	62.2 (23.4)

BMI, body mass index; SD, standard deviation. ^a^ Foot deformities were classified according to the foot with the worst deformity. Mild deformities were pes planus, pes cavus, hallux valgus, hallux limitus, hammer toes, and lesser toe amputation; moderate deformities were hallux rigidus, hallux or ray amputation, prominent metatarsal heads, and claw toes; severe deformities were Charcot deformity, (fore)foot amputation, and pes equines. ^b^ Amputations were classified according to the side with the most proximal amputation. Continuous variables are reported as mean (SD) and categorical variables as *n* (%).

**Table 2:** Area under the curve (95% confidence interval) and receiver operating characteristic (ROC) curves for the associations with the reference measure.

	**Cut-Off for “High Adherence” According to the Reference Measure**	
	**60%**	**70%**
**Proportion of steps**	1.00 (0.99–1.00)	0.98 (0.94–1.00)
**Objective wearing time**	0.99 (0.97–1.00)	0.97 (0.92–1.00)
**Subjective wearing time**	0.69 (0.52–0.85)	0.68 (0.52–0.84)
**ROC curves**	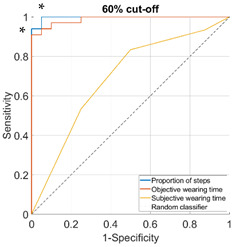	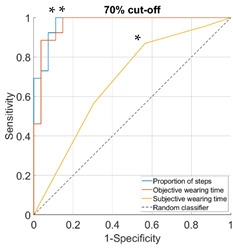
	**80%**	**90%**
**Proportion of steps**	0.96 (0.91–1.00)	0.97 (0.92–1.00)
**Objective wearing time**	0.97 (0.93–1.00)	0.89 (0.76–1.00)
**Subjective wearing time**	0.67 (0.51–0.84)	0.81 (0.66–0.95)
**ROC curves**	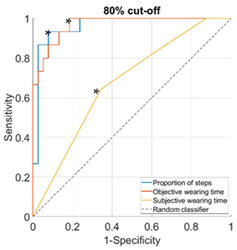	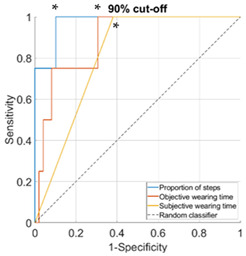

Note: diagonal lines in the ROC curves are the result of ties in the data on subjective wearing time; * suggested cut-offs, see details in Table 3.

**Table 3:** Sensitivity, specificity, positive predictive value (PPV), and negative predictive value (NPV) for different cut-offs for “high adherence” according to the reference measure.

**60% cut-off for “high adherence”**					
**Measure**	**Cut-off ^a^**	**Sensitivity**	**Specificity**	**PPV**	**NPV**
Proportion of steps	≥61.6%	100%	95%	97%	100%
Objective wearing time	≥10.5 h/day	91%	100%	100%	87%
Subjective wearing time	≥category “8–12 h/day”	83%	50%	76%	62%
**70% cut-off for “high adherence”**					
**Measure**	**Cut-off ^a^**	**Sensitivity**	**Specificity**	**PPV**	**NPV**
Proportion of steps	≥69.4.0%	100%	89%	90%	100%
Objective wearing time	≥10.5 h/day	100%	85%	87%	100%
Subjective wearing time	≥category “8–12 h/day”	87%	43%	61%	77%
**80% cut-off for “high adherence”**					
**Measure**	**Cut-off ^a^**	**Sensitivity**	**Specificity**	**PPV**	**NPV**
Proportion of steps	≥77.4%	93%	92%	82%	97%
Objective wearing time	≥12.2 h/day	100%	82%	68%	100%
Subjective wearing time	≥category “ > 12 h/day”	64%	66%	45%	81%
**90% cut-off for “high adherence”**					
**Measure**	**Cut-off ^a^**	**Sensitivity**	**Specificity**	**PPV**	**NPV**
Proportion of steps	≥86.4%	100%	90%	44%	100%
Objective wearing time	≥12.7 h/day	100%	69%	21%	100%
Subjective wearing time	≥category “ > 12 h/day”	100%	62%	20%	100%

CI, confidence interval. ^a^ The cut-off that maximized the sum of sensitivity and specificity.

## Data Availability

The datasets generated and analyzed during the current study are not publicly available due to current Dutch ethical legislation and the European Union GDPR Act.

## References

[1] Armstrong D.G., Boulton A.J.M., Bus S.A. (2017). Diabetic foot ulcers and their recurrence. N. Engl. J. Med..

[2] Bus S.A., Lavery L.A., Monteiro-Soares M., Rasmussen A., Raspovic A., Sacco I.C.N., van Netten J.J., on behalf of the International Working Group on the Diabetic Foot (IWGDF) (2020). Guidelines on the prevention of foot ulcers in persons with diabetes (IWGDF 2019 update). Diabetes/Metab. Res. Rev..

[3] Parker C.N., Van Netten J.J., Parker T.J., Jia L., Corcoran H., Garrett M., Kwok C.F., Nather A., Que M.T., Srisawasdi G. (2019). Differences between national and international guidelines for the management of diabetic foot disease. Diabetes/Metab. Res. Rev..

[4] The National Board of Health and Welfare Nationella Riktlinjer för Diabetesvård (National Guidlines for Diabetes Care). https://www.socialstyrelsen.se/globalassets/sharepoint-dokument/artikelkatalog/nationella-riktlinjer/2018-10-25.pdf.

[5] Kaminski M.R., Golledge J., Lasschuit J.W., Schott K.-H., Charles J., Cheney J., Raspovic A. (2022). Australian guideline on prevention of foot ulceration: Part of the 2021 Australian evidence-based guidelines for diabetes-related foot disease. J. Foot Ankle Res..

[6] Jarl G., Tranberg R., Johansson U., Alnemo J., Lundqvist L.-O. (2020). Predictors of adherence to wearing therapeutic footwear among people with diabetes. J. Foot Ankle Res..

[7] Arts M.L., de Haart M., Bus S.A., Bakker J.P., Hacking H.G., Nollet F. (2014). Perceived usability and use of custom-made footwear in diabetic patients at high risk for foot ulceration. J. Rehabil. Med..

[8] Waaijman R., Keukenkamp R., de Haart M., Polomski W.P., Nollet F., Bus S.A. (2013). Adherence to wearing prescription custom-made footwear in patients with diabetes at high risk for plantar foot ulceration. Diabetes Care.

[9] Connor H., Mahdi O.Z. (2004). Repetitive ulceration in neuropathic patients. Diabetes Metab. Res. Rev..

[10] Waaijman R., de Haart M., Arts M.L., Wever D., Verlouw A.J., Nollet F., Bus S.A. (2014). Risk factors for plantar foot ulcer recurrence in neuropathic diabetic patients. Diabetes Care.

[11] Jarl G., Lundqvist L.-O. (2016). Adherence to wearing therapeutic shoes among people with diabetes: A systematic review and reflections. Patient Prefer. Adherence.

[12] Ehrmann D., Spengler M., Jahn M., Niebuhr D., Haak T., Kulzer B., Hermanns N. (2018). Adherence over time: The course of adherence to customized diabetic insoles as objectively assessed by a temperature sensor. J. Diabetes Sci. Technol..

[13] Keukenkamp R., Merkx M.J., Busch-Westbroek T.E., Bus S.A. (2018). An explorative study on the efficacy and feasibility of the use of motivational interviewing to improve footwear adherence in persons with diabetes at high-risk of foot ulceration. J. Am. Podiatr. Med. Assoc..

[14] Jarl G. (2018). Methodological considerations of investigating adherence to using offloading devices among people with diabetes. Patient Prefer. Adherence.

[15] Racaru S., Saghdaoui L.B., Choudhury J.R., Wells M., Davies A.H. (2022). Offloading treatment in people with diabetic foot disease: A systematic scoping review on adherence to foot offloading. Diabetes Metab. Syndr. Clin. Res. Rev..

[16] Lazzarini P.A., Crews R.T., van Netten J.J., Bus S.A., Fernando M.E., Chadwick P.J., Najafi B. (2019). Measuring plantar tissue stress in people with diabetic peripheral neuropathy: A critical concept in diabetic foot management. J. Diabetes Sci. Technol..

[17] Bus S.A., Waaijman R., Arts M., de Haart M., Busch-Westbroek T., van Baal J., Nollet F. (2013). Effect of custom-made footwear on foot ulcer recurrence in diabetes: A multicenter randomized controlled trial. Diabetes Care.

[18] Bus S.A., Waaijman R., Nollet F. (2012). New monitoring technology to objectively assess adherence to prescribed footwear and assistive devices during ambulatory activity. Arch. Phys. Med. Rehabil..

[19] Jarl G., Tranberg R. (2017). An innovative sealed shoe to off-load and heal diabetic forefoot ulcers—A feasibility study. Diabet. Foot Ankle.

[20] Crews R.T., Shen B.J., Campbell L., Lamont P.J., Boulton A.J., Peyrot M., Kirsner R.S., Vileikyte L. (2016). Role and determinants of adherence to off-loading in diabetic foot ulcer healing: A prospective investigation. Diabetes Care.

[21] Armstrong D.G., Lavery L.A., Kimbriel H.R., Nixon B.P., Boulton A.J. (2003). Activity patterns of patients with diabetic foot ulceration: Patients with active ulceration may not adhere to a standard pressure off-loading regimen. Diabetes Care.

[22] Ababneh A., Finlayson K., Edwards H., Lazzarini P.A. (2022). Factors associated with adherence to using removable cast walker treatment among patients with diabetes-related foot ulcers. BMJ Open Diabetes Res. Care.

[23] Crews R.T., Armstrong D.G., Boulton A.J. (2009). A method for assessing off-loading compliance. J. Am. Podiatr. Med. Assoc..

[24] Najafi B., Crews R.T., Wrobel J.S. (2010). Importance of time spent standing for those at risk of diabetic foot ulceration. Diabetes Care.

[25] Lutjeboer T., Postema K., Hijmans J. (2018). Validity and feasibility of a temperature sensor for measuring use and non-use of orthopaedic footwear. J. Rehabil. Med..

[26] Menz H.B., Bonanno D.R. (2021). Objective measurement of adherence to wearing foot orthoses using an embedded temperature sensor. Med. Eng. Phys..

[27] Jarl G., Alnemo J., Tranberg R., Lundqvist L.-O. Predictors of Adherence to Using Therapeutic Shoes among People with Diabetic Foot Complications. Proceedings of the 8th International Symposium on the Diabetic Foot.

[28] Van Netten J.J., Hijmans J.M., Jannink M.J., Geertzen J.H., Postema K. (2009). Development and reproducibility of a short questionnaire to measure use and usability of custom-made orthopaedic shoes. J. Rehabil. Med..

[29] Schaper N.C., van Netten J.J., Apelqvist J., Bus S.A., Hinchliffe R.J., Lipsky B.A. (2020). Practical Guidelines on the prevention and management of diabetic foot disease (IWGDF 2019 update). Diabetes/Metab. Res. Rev..

[30] Mills J.L., Conte M.S., Armstrong D.G., Pomposelli F.B., Schanzer A., Sidawy A.N., Andros G., Society for Vascular Surgery Lower Extremity Guidelines Committee (2014). The society for vascular surgery lower extremity threatened limb classification system: Risk stratification based on wound, ischemia, and foot infection (WIfI). J. Vasc. Surg..

[31] Fokkenrood H., Verhofstad N., Van Den Houten M., Lauret G., Wittens C., Scheltinga M., Teijink J. (2014). Physical activity monitoring in patients with peripheral arterial disease: Validation of an activity monitor. Eur. J. Vasc. Endovasc. Surg..

[32] Van Schooten K.S., Rispens S.M., Elders P.J., Lips P., van Dieën J.H., Pijnappels M. (2015). Assessing physical activity in older adults: Required days of trunk accelerometer measurements for reliable estimation. J. Aging Phys. Act..

[33] Matthews C.E., Ainsworth B.E., Thompson R.W., Bassett D.R. (2002). Sources of variance in daily physical activity levels as measured by an accelerometer. Med. Sci. Sports Exerc..

[34] Dijkstra B., Kamsma Y., Zijlstra W. (2010). Detection of gait and postures using a miniaturised triaxial accelerometer-based system: Accuracy in community-dwelling older adults. Age Ageing.

[35] Storm F.A., Heller B.W., Mazzà C. (2015). Step detection and activity recognition accuracy of seven physical activity monitors. PLoS ONE.

[36] Schober P., Boer C., Schwarte L.A. (2018). Correlation coefficients: Appropriate use and interpretation. Anesth. Analg..

[37] McHugh M.L. (2012). Interrater reliability: The kappa statistic. Biochem. Med..

[38] Mandrekar J.N. (2010). Receiver operating characteristic curve in diagnostic test assessment. J. Thorac. Oncol..

[39] Price P. (2016). How can we improve adherence?. Diabetes Metab. Res. Rev..

[40] Jarl G., van Netten J.J., Lazzarini P.A. (2023). Fragile feet and trivial trauma: Communicating etiology of diabetes-related foot ulcers with patients. J. Am. Podiatr. Med. Assoc..

[41] Bus S.A., Armstrong D.G., Gooday C., Jarl G., Caravaggi C., Viswanathan V., Lazzarini P.A., On behalf of the International Working Group on the Diabetic Foot (IWGDF) (2020). Guidelines on offloading foot ulcers in persons with diabetes (IWGDF 2019 update). Diabetes Metab. Res. Rev..

